# Rifampin Pharmacokinetics/Pharmacodynamics in the Hollow-Fiber Model of Mycobacterium kansasii Infection

**DOI:** 10.1128/aac.02320-21

**Published:** 2022-03-22

**Authors:** Shashikant Srivastava, Gunavanthi D. Boorgula, Jann-Yuan Wang, Hung-Ling Huang, Dave Howe, Tawanda Gumbo, Scott K. Heysell

**Affiliations:** a Department of Pulmonary Immunology, University of Texas Health Science Center, Tyler, Texas, USA; b Department of Immunology, UT Southwestern Medical Center, Dallas, Texas, USA; c Department of Pharmacy Practice, Texas Tech University Health Science Center, Dallas, Texas, USA; d Department of Internal Medicine, National Taiwan University Hospital, Taipei, Taiwan; e Department of Internal Medicine, Kaohsiung Medical University Hospital, Kaohsiung, Taiwan; f Graduate Institute of Medicine, Kaohsiung Medical University, Kaohsiung, Taiwan; g Quantitative Preclinical & Clinical Sciences Department, Praedicare, Inc., Dallas, Texas, USA; h Department of Medicine, University of Cape Town, Observatory, South Africa; i Division of Infectious Diseases and International Health, University of Virginia, Charlottesville, Virginia, USA

**Keywords:** nontuberculous mycobacteria, rifampin, hollow-fiber model system

## Abstract

There is limited high-quality evidence to guide the optimal treatment of Mycobacterium kansasii pulmonary disease. We retrospectively collected clinical data from 33 patients with M. kansasii pulmonary disease to determine the time-to-sputum culture conversion (SCC) upon treatment with a standard combination regimen consist of isoniazid-rifampin-ethambutol. Next, MIC experiments with 20 clinical isolates were performed, followed by a dose-response study with the standard laboratory strain using the hollow-fiber system model of M. kansasii infection (HFS-*Mkn*). The inhibitory sigmoid maximum effect (*E*_max_) model was used to describe the relationship between the bacterial burden and rifampin concentrations. Finally, *in silico* clinical trial simulations were performed to determine the clinical dose to achieve the optimal rifampin exposure in patients. The SCC rate in patients treated with combination regimen containing rifampin at 10 mg/kg of body weight/day was 73%, the mean time to SSC was 108 days, and the mean duration of therapy was 382 days. The MIC of the M. kansasii laboratory strain was 0.125 mg/L, whereas the MICs of the clinical isolates ranged between 0.5 and 4 mg/L. In the HFS-*Mkn* model, a maximum kill (*E*_max_) of 7.82 log_10_ CFU/mL was recorded on study day 21. The effective concentration mediating 80% of the *E*_max_ (EC_80_) was calculated as the ratio of the maximum concentration of drug in serum for the free, unbound fraction (*fC*_max_) to MIC of 34.22. The target attainment probability of the standard 10-mg/kg/day dose fell below 90% even at the MIC of 0.0625 mg/L. Despite the initial kill, there was M. kansasii regrowth with the standard rifampin dose in the HFS-*Mkn* model. Doses higher than 10 mg/kg/day, in combination with other drugs, need to be evaluated for better treatment outcome.

## INTRODUCTION

Clinically significant nontuberculous mycobacteria (NTM) are a growing burden on a global level, with an increasing number of mycobacterial species described (1) and a greater number of reported infections (2). Mycobacterium kansasii is one of the most virulent and prevalent NTM species and one of the six most frequently isolated NTM species across the world. The clinical presentation of M. kansasii pulmonary disease mimics classical tuberculosis (TB) diseases caused by Mycobacterium tuberculosis (3).

The standard treatment of M. kansasii pulmonary disease is a combination regimen extrapolated from TB treatment. The drugs in the standard combination regimen are isoniazid (INH) (5 mg/kg of body weight/day), rifampin (10 mg/kg of body weight/day), and ethambutol (15 mg/kg of body weight/day). The recent multisociety NTM treatment guideline recommends daily or intermittent therapy when a macrolide-based regimen is used and daily therapy when an INH-based regimen is used (4). The 2020 guidelines also recommend that the M. kansasii infection could be treated for a fixed duration of 12 months instead of 12 months beyond culture conversion (4).

Rifampin is one of the key drugs in the M. kansasii combination therapy, and resistance to rifampin has been associated with treatment failure (5, 6). One clinical study published way back in 1981, which included 256 patients to evaluate the efficacies of different drugs for the treatment of M. kansasii pulmonary diseases, reported the chances of therapy failure were higher with regimens without rifampin (7). Unlike for M. tuberculosis, there is a lack of pharmacokinetic/pharmacodynamic (PK/PD) studies conducted to optimize the dose of rifampin as well as other drugs in the combination therapy for the treatment of M. kansasii pulmonary disease. There are also no randomized control trials that have been conducted to associate the baseline MIC of the infecting strains with the clinical outcome.

Pulmonary M. kansasii infection is classified as a rare disease, which makes it difficult to establish an evidence base for therapeutic decision-making (https://rarediseases.info.nih.gov/diseases). Therefore, to fill in the knowledge gap on the optimal dose of the drugs in the standard regimen, it is essential to perform monotherapy PK/PD experiments to determine the optimal dose of each drug, followed by experiments comparing the efficacy of the optimal dose combination with the standard of care regimen for efficacy and resistance suppression. Therefore, the aim of the present study was (i) to determine the time to negative sputum culture conversion (SCC) and therapy duration in patients treated with the standard combination regimen for M. kansasii pulmonary disease, (ii) to perform rifampin susceptibility testing and determine MICs of 20 M. kansasii clinical strains, (iii) to perform PK/PD studies using the preclinical hollow-fiber system model (8–10) of M. kansasii infection (HFS-*Mkn*) to determine the optimal rifampin exposure for M. kansasii kill, and (iv) to determine the target attainment probability (TAPs) of different clinical doses of rifampin to achieve the optimal exposure target, as identified in the HFS-*Mkn* model, across the MIC range in clinical strains. We propose that the rifampin optimal dose combination regimen will result in successful treatment outcome and also could possibly shorten the therapy duration for M. kansasii pulmonary disease.

## RESULTS

We used the data from a retrospective study to benchmark the efficacy of the standard regimen containing 10 mg/kg/day rifampin. There were 33 patients fulfilling the criteria for M. kansasii pulmonary disease and treated with the standard combination regimen (consisting of isoniazid, rifampin, and ethambutol) and who had serial sputum samples collected during the therapy. The baseline patient demographics, clinical characteristics, sputum culture conversion (SCC), and therapy duration are summarized in Table 1. The mean time-to-negative sputum culture in these 33 patients was 100 days (range, 17 to 406 days), and the mean duration of therapy was 372 days (range, 35 to 657 days). Out of these 33 patients, no SCC was reported in 10 (30.3%) patients, hence indicating failure of the combination therapy. The MIC of any of the drug in the regimen was not available in the records to report and to conclude if emergence of drug resistance was the cause of therapy failure.

**TABLE 1:** Demographics and clinical characteristics of 33 Taiwanese patients

Parameter	Result for^*a*^:		
	Males	Females	Total
Patient demographics			
No. (%)	25 (76)	8 (24)	
Age, yr (range)	60 (28–79)	75 (55–89)	
Wt, kg (range)	60 (31–89)	43 (31–57)	
Chest X-ray score^*a*^	8 (1–15)	8 (3–13)	

Therapy^*b*^			
SCC, no. (%)	17 (68)	6 (75)	23/33 (69.7)
Mean time to SCC, days (range)	95 (21–406)	115 (17–357)	100 (17–406)
Mean therapy duration, days (range)	385 (187–657)	333 (35–635)	372 (35–657)

a The chest X-ray score was noted as previously described (36). Briefly, each lung was divided into 3 areas. The extent of infiltration in each area was rated on a 4-point scale of 0 to 3, with a maximum score of 18.

b Sputum culture conversion (SCC) results are shown for patients (*n* = 33) administered a mean RIF dose of 10.1 mg/kg (range, 6.6 to 14.5 mg/kg).

**FIG 1:**
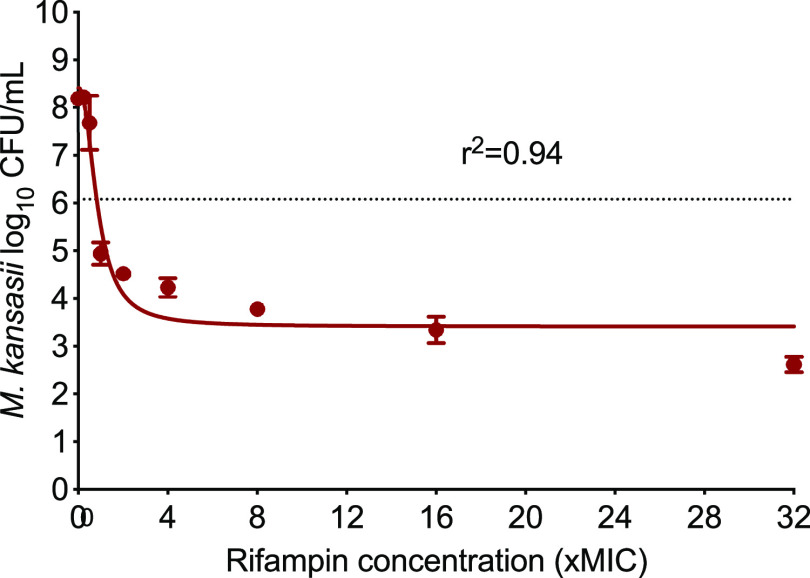
Rifampin concentration response in test tubes. The dotted line represents the stasis or starting inoculum. As shown in the figure, compared to the nontreated control (8.41 ± 0.27 log_10_ CFU/mL), the highest rifampin concentration of 32×MIC (or 256 mg/L) killed 5.00 ± 0.34 log_10_ CFU/mL M. kansasii cells in 7 days.

The rifampin MIC of the laboratory strain was 0.125 mg/L. Table 2 shows the rifampin MIC distribution in the 20 clinical strains, where the MIC_50_ and MIC_90_ were calculated as 0.5 and 4 mg/L, respectively. Figure 1 shows the results of the rifampin dose-response study performed in the test tubes at static concentrations, where the inhibitory sigmoid maximum effect (*E*_max_) model was used to describe the relationship between the bacterial burden and drug concentration (expressed as “×MIC”). Compared to the nontreated controls, rifampin killed 5.00 ± 0.34 log_10_ CFU/mL M. kansasii in 7 days. The rifampin concentration mediating 50% of the maximal kill *E*_max_ (EC_50_) was calculated as 0.88×MIC, or 0.11 mg/L.

**TABLE 2:** Rifampin MIC distribution among M. kansasii clinical strains

Clinical strain ID and MIC_50_ or MIC_90_	Rifampin MIC (mg/L)
Strains	
MRN2392724	1
MRN2739081	2
18:58688	0.5
MK_881	1
MK_915	0.25
MK_918	0.5
MK_860	0.5
MK_806	0.5
MK_978	0.5
MK_976	0.5
MK_925	4
MK_887	2
MK_829	4
MK_902	0.5
MK_817	0.5
MK_826	0.5
MK_997	4
MK_930	0.5
MK_1000	1
MK_1005	4

MIC_50_ or MIC_90_	
MIC_50_	0.5
MIC_90_	4

**FIG 2:**
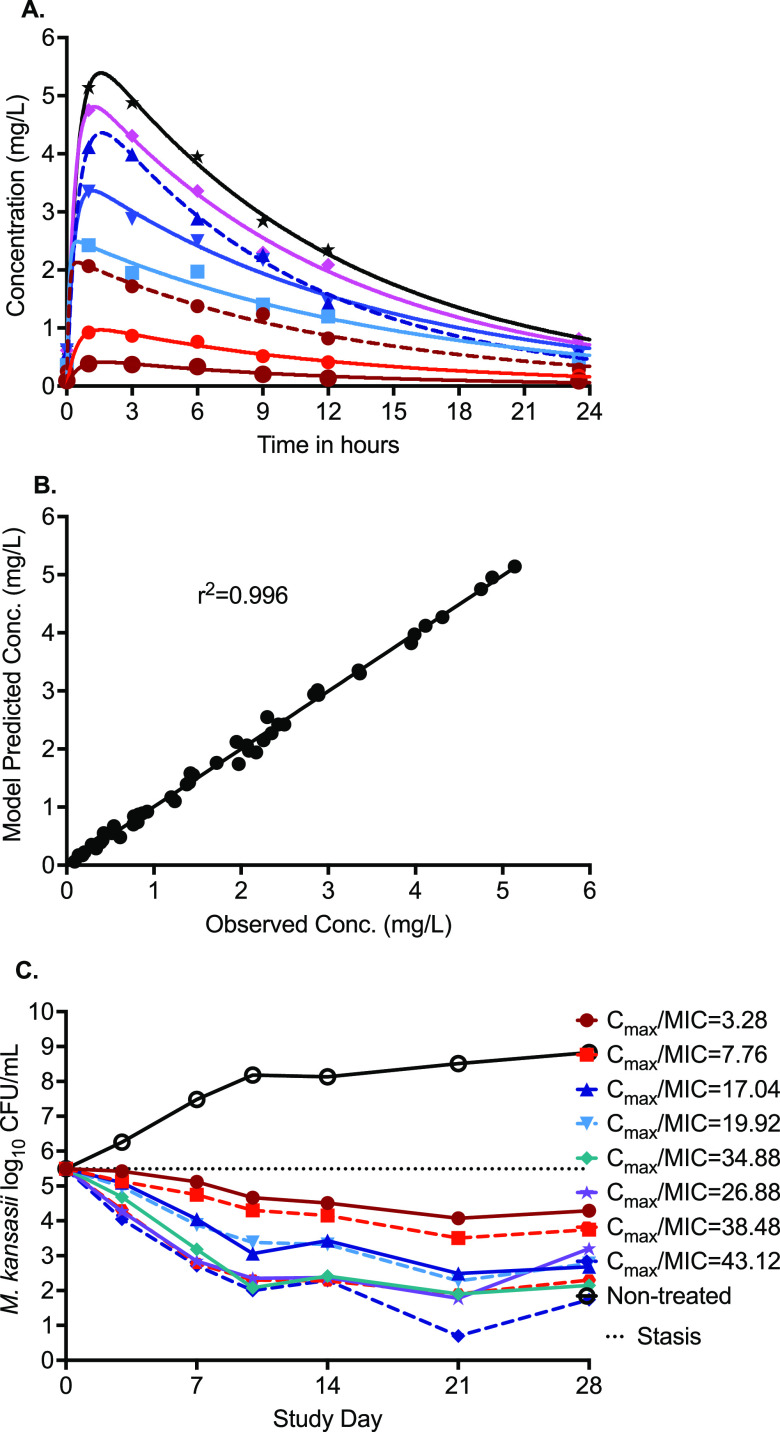
Rifampin pharmacokinetics and bacteria kill curves in the HFS-*Mkn* model. (A) Concentration-time profile of different rifampin doses, where the solid line represents the model predicted concentrations and symbols represent the measured drug concentrations in the HFS-*Mkn* samples. (B) Model fit with an *r*^2^ value of 0.996 showing minimal bias between the pharmacokinetics model predicted versus measured drug concentration. (C) The extent of bacterial kill in the HFS-*Mkn* model varied in a dose-dependent manner. All eight rifampin doses were able to keep the bacterial burden below stasis during the 28 days of study.

The concentration-time profiles of different rifampin doses in the HFS-*Mkn* study are shown in Fig. 2A. In this HFS-*Mkn* study, the rifampin half-life was calculated as 6.09 h (range, 2.41 to 7.72 h), which was indeed longer than the intended 3-h half-life; however, it was still in the range of that reported in clinical trials (median, 2.30 h; range, 1.12 to 10.45 h) (11). Among the other PK parameters, the rifampin clearance in the HFS-*Mkn* model was calculated as 23.02 L/h (95% confidence interval [CI], 21.40 to 24.65), and the volume of distribution was calculated as 285.8 L (95% CI, 239.6 to 332). Figure 2B shows the goodness-of-fit plot for the PK model predicted concentrations versus the HFS-*Mkn* model’s measured drug concentrations. The measured drug concentrations were used to calculate ratio of the the maximum concentration of drug in serum for the free, unbound fraction of drug (*fC*_max_) to MIC and area under the concentration-time curve from 0 to 24 h for the free, unbound fraction of drug (*f*AUC_0–24_)/MIC with each of the eight rifampin doses, as summarized in Table 3.

**TABLE 3:** Rifampin exposures achieved in the HFS-*Mkn* model and bacterial burden at time points when maximal growth and therapy failure were reported^*a*^

Regimen ID	*fC*_max_/MIC ratio	*f*AUC_0–24_/MIC ratio	Log_10_ CFU/mL at:	
			Day 21	Day 28
Nontreated	0	0	8.512	8.825

Treated				
R1	3.28	42.16	4.072	4.288
R2	7.76	107.6	3.505	3.7482
R3	17.04	225.36	2.491	2.681
R4	19.92	310.4	2.279	2.778
R6	34.88	399.68	1.903	2.146
R5	26.88	400.16	1.778	3.204
R7	38.48	499.92	1.903	2.301
R8	43.12	568.72	0.699	1.7404

a The MIC of M. kansasii ATCC 12478 is 0.125 mg/L.

**FIG 3:**
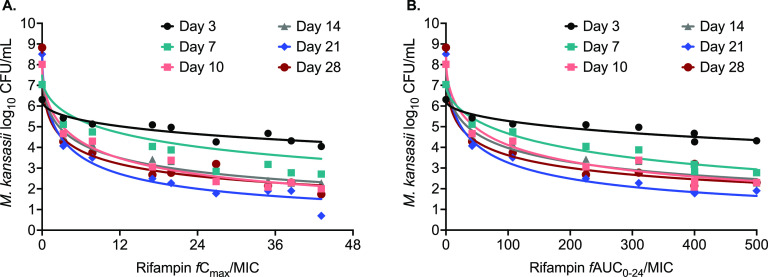
Rifampin dose response in the HFS-*Mkn* model. Shown is the relationship between the drug dose and bacterial burden in terms of (A) the *fC*_max_/MIC ratio or (B) the *f*AUC_0–24_/MIC ratio, using the inhibitory sigmoid *E*_max_ model. Based on the highest *r*^2^ value and lowest AICc score, the *fC*_max_/MIC ratio was determined as the PK/PD index linked to efficacy against M. kansasii.

The changes in the number of viable THP-1 cells in the HFS-*Mkn* model over the 28-day study period are shown in Fig. S1 in the supplemental material. Figure 2C shows the bacterial kill curve with each rifampin dose, using the CFU/mL readouts, in the HFS-*Mkn* model over 28 days of the study. The extent of bacterial kill was dose dependent, and the maximum kill of 7.81 log_10_ CFU/mL compared to the nontreated controls was recorded on day 21 of the study. The relationships between the CFU and rifampin concentration (*fC*_max_/MIC ratio and *f*AUC_0–24_/MIC ratio) are shown in Fig. 3A and B. The highest *r*^2^ value and lowest Akaike information criterion score (AICc) (12) were used to select the PK/PD index linked to rifampin efficacy against M. kansasii in the HFS-*Mkn* model. On study day 21, the *r*^2^ value and AICc score for the *fC*_max_/MIC ratio were 0.988 and 10.12, respectively, whereas the *r*^2^ value and AICc score for the *f*AUC_0–24_/MIC ratio were 0.975 and 10.57, respectively. Therefore, the *fC*_max_/MIC ratio was selected as the PK/PD index linked to the efficacy of rifampin in the HFS-*Mkn* model.

On day 21 when the maximal kill was recorded, the rifampin EC_50_ was calculated as an *fC*_max_/MIC ratio of 3.53 with an *H* of 0.61 (*r*^2^ = 0.99). This translates to an EC_80_ or optimal *fC*_max_/MIC ratio for M. kansasii kill as 34.22. Regarding the emergence of rifampin resistance in the HFS-*Mkn* model, although we noticed the change in the kill curve trajectories on study day 28, there was no rifampin-resistant subpopulation recorded on agar supplemented with 3 mg/L rifampin. Since, 3 mg/L corresponds to 24×MIC of the ATCC strain used in the HFS-*Mkn* study, we performed a MIC experiment using the day-28 MGIT-positive cultures (see Fig. S2 in the supplemental material). There was no change in the rifampin MIC in the nontreated control systems as well as in the culture from HFS-*Mkn* model treated with the different rifampin. The results of the MGIT-derived time-to-positive (TTP) readouts, as the second pharmacodynamics measure, are shown in Fig. S3 in the supplemental material.

**FIG 4:**
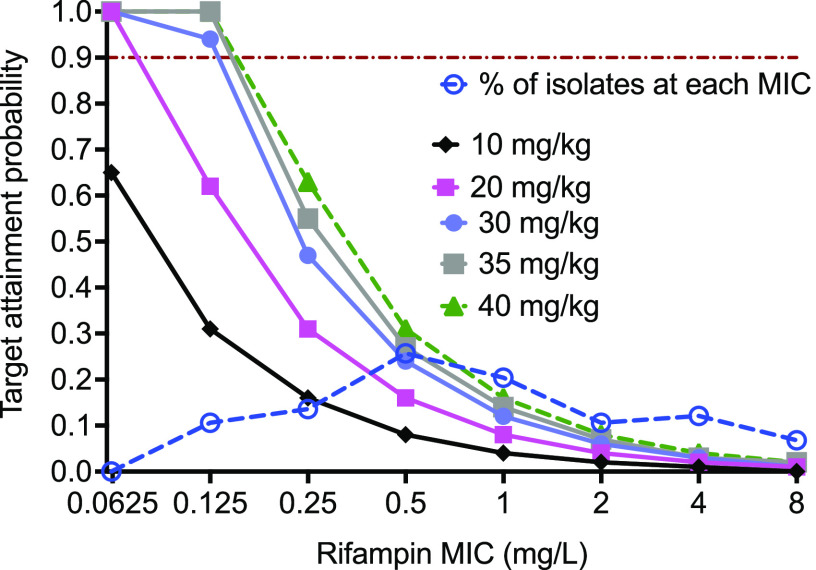
*In silico* simulations of different rifampin doses for PK/PD target attainment. The target attainment probability of different rifampin doses for the probability to achieve an *fC*_max_/MIC ratio of 34.22 was calculated. All simulated doses failed to achieve the target attainment in 90% of the patients beyond a MIC of 0.125 mg/L.

The target attainment probability (TAP) of different rifampin clinical doses, ranging from 10 mg/kg (i.e., 600 mg/day) to 40 mg/kg (i.e., 2,400 mg/day) to achieve the optimal exposure target of an *fC*_max_/MIC ratio of 34.22 was simulated using the input population PK data from the literature (13). Figure 4 shows the TAP with five simulated rifampin doses, summated over the range of MICs in the clinical strains. To put the results into clinical context, the TAP of the 10 mg/kg/day or 600-mg/daily dose was only 65%, even at the lowest MIC of 0.0625 mg/L used in the simulations. Higher-than-standard rifampin doses were predicted to have better performance; however, the TAP fell below 90% even with the dose of 40 mg/kg/day, and the susceptibility breakpoint for this dose was determined as 0.25 mg/L.

## DISCUSSION

Combination therapy is the standard of care for the treatment of M. kansasii pulmonary disease, where rifampin is one of the key drugs. While prior studies show high failure rates of the combination regimen (14, 15), these studies could not establish the correlation between the *in vitro* rifampin resistance and clinical outcome. Moreover, the doses for M. kansasii were never optimized and rather were carried forward from those used to treat M. tuberculosis infections. This reemphasizes our hypothesis that the dose of each drug in the combination regimen should be optimized for each specific pathogen—in this case M. kansasii—while considering the MIC of the clinical strains as well as synergy/antagonism between the drug pairs.

In the present study, we first show the clinical response (albeit in a limited number of patients) of the rifampin-containing standard combination regimen for the treatment of M. kansasii pulmonary infections. The SCC rate in the patients treated with 10 mg/kg/day (equivalent to 600 mg daily) was only 73%, the mean time to SCC was 108 days, and the mean duration of therapy was 382 days. While only 33 patients were included in the study, these findings still reinforce that higher-than-recommended standard doses of the drugs, including rifampin, are required for a successful treatment outcome and lend credence to the aim that a higher dose combination may lead to shorter duration of therapy than the currently recommended 12 months (4).

Second, we show that similar to M. tuberculosis studies (11, 16, 17), the rifampin PK/PD index linked to efficacy was the ratio of *C*_max_ to MIC. We found an *fC*_max_/MIC ratio of 34.22 as the optimal PK/PD exposure target for the treatment of M. kansasii pulmonary disease. Since the efficacy of rifampin against M. kansasii is linked to the ratio of *C*_max_ to MIC, the probability of achieving the optimal exposure target with a given dose will decrease with an increase in the MIC. Considering the MIC distribution of rifampin in the clinical strains (18) and the TAP with 30-, 35-, and 40-mg/kg/day doses were not different, we propose a 30-mg/kg/day (2,400-mg/day) clinical dose to be tested in the patients, which could also help reduce the duration of therapy. While rifampin doses up to 50 mg/kg/day have been tested for the treatment of TB (19), given that the duration of therapy of M. kansasii pulmonary disease is longer than the 6-month short-course therapy duration of pulmonary TB and the patient population may be older or have more comorbidities than people with TB, carefully designed studies should include rigorous adverse event monitoring and consideration of variable durations beyond SCC.

Our study has limitations. None of the 33 patients we included in the retrospective study were living with HIV. In immunocompromised patients, such as those living with HIV, or patients with other advanced pulmonary diseases are more prone to M. kansasii disease, and the response to the therapy may be different. Furthermore, drug interactions of some antiretroviral therapy or other immunomodulatory therapies with rifampin may preclude its use (20). Next, while we noticed the rifampin monotherapy failure in the HFS-*Mkn* model on study day 28, we failed to capture any drug-resistant subpopulation. The possible reasons could be use of an M. kansasii laboratory strain with a low MIC or the high drug concentration in the agar, or perhaps the drug-resistant subpopulation was simply below the limits of detection (0.69 log_10_ CFU/mL) of the agar-based culture methods. It could also be that we used a single phenotypic method for resistance determination and did not assay the *rpoB* resistance-determining region, where mutations may have been observed in the absence of phenotypic resistance (21). Finally, while we showed the optimal exposure target could not be achieved with the standard rifampin dose, the treatment of M. kansasii pulmonary disease is always a combination therapy, where other drugs may have synergy with rifampin. Therefore, combination regimens with rifampin at a lower-than-optimal dose could still kill M. kansasii. Such drug interaction studies are in progress to determine the optimal dose of each drug in the regimen alone and in combination.

To summarize, in the HFS-*Mkn* model, the rifampin optimal exposure target for M. kansasii kill was an *fC*_max_/MIC ratio of 34.22, which cannot be achieved with the currently recommended 10 mg/kg/day clinical dose. We propose to test the 30-mg/kg/day dose in the combination regimen for a better therapy outcome and the possibility to shorten the duration of therapy for M. kansasii pulmonary disease.

## MATERIALS AND METHODS

### Clinical data collection.

To benchmark the efficacy of currently recommended treatment regimen, we performed a retrospective study to collect clinical data from a subset from a previously published study (22) with Taiwanese patients undergoing treatment for M. kansasii pulmonary disease between 2008 and 2014. The patients who fulfilled the composite diagnostic criteria for M. kansasii pulmonary disease were identified and treated with standard combination regimen consist of isoniazid plus rifampin plus ethambutol, as recommended by the American Thoracic Society (4, 23). Serial sputum samples from each patient were collected to determine the bacterial growth using the mycobacterial growth indicator tube (MGIT) automated liquid culture system. Information on patient demographics and HIV infection status was collected from the case report forms (10, 22). Hospital charts were also scanned to record the signs, symptoms, medical history, and other laboratory data to exclude patients with diagnoses of diseases other than M. kansasii pulmonary disease. The study protocol was approved by the institutional ethic committees of National Taiwan University Hospital and Kaohsieng Medical University Hospital (NTUH-REC-201508017RIND and KMUHIRB-SV[I]-2015200266).

### Bacteria, cell lines, drugs, and other supplies.

The M. kansasii standard laboratory strain (ATCC 12478) and 20 clinical strains (separate from the 33 patients in the retrospective study) were used in the experiments. Middlebrook 7H9 broth and Middlebrook 7H10 agar, both supplemented with 10% oleic acid-albumin-catalase-dextrose (OADC), were used to culture the bacteria (8, 24). The growth medium for the human monocyte-derived THP-1 cell line (ATCC TIB-202) was RPMI 1640 supplemented with 10% fetal bovine serum (FBS). In the HFS-*Mkn* studies, the circulating medium was RPMI 1640 supplemented with 2% FBS. Rifampin was purchased from Sigma-Aldrich (St. Louis, MO), polyvinylidene difluoride (PVDF) hollow-fiber cartridges were purchased from FiberCell Systems, Inc. (MD, USA), and the MGIT liquid culture system and EpiCenter software were purchased from Becton Dickinson, USA.

### MIC experiment.

The MIC of rifampin was determined using the broth microdilution method (25). The MIC experiments were performed twice with three replicates per drug concentration. Briefly, before each experiment, cultures (strain ATCC 12478 and 20 clinical strains) were grown to the logarithmic phase in Middlebrook 7H9 broth supplemented with 10% OADC. The turbidity of the cultures was adjusted to a McFarland standard of 0.5, followed by 100-fold dilution with the intent to get a bacterial density of ∼1.5 × 10^5^ CFU/mL. Next, 180 μL of the inoculum was added to each of the 96 wells prefilled with 20 μL of each rifampin concentration (10×), ranging between 0.125 and 64 mg/L. After 7 days of incubation at 37°C, cultures were visually inspected using an inverted mirror. The lowest rifampin concentration that completely inhibited M. kansasii growth (absence of bacterial pellet) was determined as the MIC.

### Rifampin time-kill study at static concentration.

The experiment was performed only with the standard laboratory strain ATCC 12478, with inoculum preparation and drug concentration range as described above. The total culture volume was 5 mL. After 7 days of drug exposure, a 1-mL sample from each test tube was collected, washed twice to remove carryover drug, and serially diluted 10-fold before inoculation on Middlebrook 7H10 agar supplemented with 10% OADC. The M. kansasii colonies were counted after 10 days of incubation at 37°C. The relationship between the different rifampin concentrations and the M. kansasii bacterial burden (log_10_ CFU/mL) was described using the inhibitory sigmoid *E*_max_ model.

### Rifampin pharmacokinetics/pharmacodynamics in the HFS-*Mkn* model.

Elsewhere we have published a detailed description of the HFS model in general (26, 27) and how to perform the PK/PD studies with M. kansasii (8, 10, 21, 24). In the present study, the same HFS-*Mkn* model was used without any modification. The dilution rate in the HFS-*Mkn* model was set to mimic the rifampin pharmacokinetics achieved in patients (16, 28, 29), with the intent to simulate a 3-h half-life. Rifampin has 80% protein binding: in other words, the free (*f*) drug concentration to exert the antimycobacterial effect is 20% of the total rifampin concentration (29, 30). Prior population PK studies report that a 10-mg/kg/day dose (equivalent to 600 mg daily) results in a serum AUC_0–24_ of 30.7 ± 13.2 mg · h/L (*f*AUC_0-24_, 6.14 ± 2.64) in HIV-negative patients (13), whereas the AUC_0–24_ was 62.61 + 2.44 mg · h/L (*f*AUC_0-24_ 12.55 ± 0.08) in HIV-infected patients. Thus, since it is the free or non-protein-bound fraction of a drug that is pharmacologically active (31, 32), all rifampin doses tested in the HFS-*Mkn* model represent the free drug concentration, where we assumed a linear relationship between the rifampin dose and resulting free drug concentration (28, 29). We tested eight different rifampin doses, including the standard dose of 10 mg/kg/day, as well as a high dose of up to 50 mg/kg/daily, shown to yield increased bactericidal activity in patients with TB (19).

To briefly describe the infection of the HFS-*Mkn* model and sampling of the systems, the THP-1 cells were infected with log-phase-growth M. kansasii cultures at a multiplicity of infection (MOI) of 1:1. After 4 h of infection, THP-1 cells were washed twice with warm RPMI 1640 to remove the extracellular bacteria. Next, 20 mL of the infected THP-1 cells was inoculated into the peripheral compartment of each of the 10 HFS-*Mkn* units. The circulating medium was RPMI 1640 supplemented with 2% FBS. The HFS-*Mkn* units were treated with different rifampin doses, where the drug was administered over 1 h into the central compartment. The drug infusion rate and time were controlled using computerized syringe pumps, and 1st order kinetics was used to explain the drug diffusion into the peripheral compartment (across the hollow fibers into and out of the extracapillary space). To study the steady-state PK of each rifampin dose, on day 7 of the study, the central compartment of each HFS-*Mkn* unit was sampled predose, followed by 1, 3, 6, 9, 12, and 23.5 h post-drug infusion. For the pharmacodynamics of rifampin in the HFS-*Mkn* model, samples were collected from the peripheral compartment of each HFS-*Mkn* unit on days 3, 7, 10, 14, 21, and 28. The number of viable THP-1 cells in each HFS-*Mkn* unit was determined using an automated cell counter (Scepter 2.0 cell counter; Millipore Sigma) as well as by manual counting with a hemocytometer. The carryover drug in the samples was removed by washing twice with normal saline, followed by 10-fold serial dilution to determine the bacterial burden using the Middlebrook 7H10 agar supplemented with 10% OADC. The same samples were also cultured on agar supplemented with 3 mg/L rifampin to determine the drug-resistant subpopulation at each time point. The CFU were recorded after 10 days of incubation at 37°C. As a second pharmacodynamics method, we used the MGIT liquid culture system, because it is more sensitive than the solid agar culture methods. Five hundred microliters of the washed and undiluted sample from each HFS-*Mkn* unit was inoculated into the MGIT tubes. EpiCenter software was used to record the growth units and time to positive in each sample.

In addition, on day 28, we performed MIC experiments with bacteria grown in the MGIT tubes, to determine the actual change in the MIC using a colorimetric resazurin assay (8). Briefly, a turbidity-adjusted inoculum was prepared as described above and dispensed to the 96-well plates prefilled with different rifampin concentrations. After 7 days of incubation at 37°C, 20 μL of the resazurin dye (final concentration, 0.001% [vol/vol]) was added to each well, and cultures were incubated for an additional 24 h to record the color change. The drug concentration in the well with no visible bacterial pellet as well as no color change from blue to pink was recorded as the MIC.

### Measurement of rifampin concentration and PK/PD analysis.

The method to measure the rifampin concentration in the experimental samples was published previously and was used without any modification (10, 21, 33, 34). Briefly, liquid chromatography-tandem mass spectrometry (LC-MS/MS) analysis was performed using a Waters Acquity ultraperformance liquid chromatography (UPLC) device coupled with a Waters Xevo TQ mass spectrometer. Separation was achieved by injecting 10 μL of sample on a Waters Acquity UPLC HSS T3 column (50 by 2.1 mm, 1.8 μm) using a binary gradient. The following solvents were used for UPLC: solvent A was 0.1% aqueous formic acid, and solvent B was 0.1% formic acid in methanol. Samples were diluted 1:10 with an internal standard solution containing rifampin-d3. The transitions used were *m*/*z* 823 to 791 for rifampin and *m*/*z* 826 to 794 for rifampin-d3. The between-day percentage of coefficient of variation (%CV) for analysis of low-quality controls (with high-quality controls in parentheses) controls were 8% (8%). The intraday %CV for rifampin was 8% (6%). The lower limit of quantitation was 0.01 μg/mL.

Measured rifampin concentrations in the HFS-*Mkn* model were modeled using a one-compartment model with first-order input and elimination and used to calculate the peak concentration (*C*_max_) and AUC_0–24_, as well as the half-life, clearance rate, and volume of distribution, by using Phoenix WinNonLin (35). The four-parameter inhibitory sigmoid *E*_max_ model was used to determine the relationship between rifampin exposure and bacterial burden (log_10_ CFU/mL). The exponential-growth model was used on the MGIT-derived time-to-positive readouts (the lower the bacterial burden, the higher the time-to-positive value). We used GraphPad Prism (v8) for graphing the data.

### *In silico* simulation for target attainment probability of different rifampin doses.

To identify the minimal dose of rifampin best able to achieve or exceed the EC_80_ concentration, we performed clinical trial simulations with 10,000 virtual patients. The population PK parameter input estimates were those identified by Wilkins et al. (13). We utilized a clearance of 19.2 L/h (with interindividual variability as %CV [IIV] of 0.32), the volume of 52.8 L (IIV = 0.43), and absorption constant of 1.61 h^−1^ (IIV = 2.39) (13). The rifampin protein binding of 80% was taken into account (29, 30). The target attainment probability, which is how well a dose of 600 mg (10 mg/kg/day), 1,200 mg (20 mg/kg/day), 1,800 mg (30 mg/kg/day), 2,100 mg (35 mg/kg/day), or 2,400 mg (40 mg/kg/day) would achieve the EC_80_ in the lung of patients with M. kansasii pulmonary disease, at each MIC ranging from 0.0625 mg/L to 4.0 mg/L, was then calculated. We utilized MIC distributions of rifampin among our 20 clinical isolates, as well as 132 clinical isolates, as reported in the study by Litvinov et al. (18).
