# Supplementary material for: Rifampin Pharmacokinetics/Pharmacodynamics in the Hollow-Fiber Model of Mycobacterium kansasii Infection
Source: Antimicrob Agents Chemother. 2022 Mar 22;66(4):e02320-21. doi: 10.1128/aac.02320-21 (PMC9017304; doi:10.1128/aac.02320-21)
Supplement: Supplemental file 1 — Fig. S1 to S3. Download aac.02320-21-s0001.pdf, PDF file, 1.1 MB [file aac.02320-21-s0001.pdf]

Rifampin pharmacokinetics/pharmacodynamics in the hollow fiber model of *Mycobacterium kansasii*

Shashikant Srivastava[1,2,3*], Gunavanthi D. Boorgula[1], Jann-Yuan Wang[4], Hung-Ling Huang[5,6], Dave Howe[7], Tawanda Gumbo[7,8], Scott K Heysell[9]

[1]Department of Pulmonary Immunology, University of Texas Health Centre, Tyler, Texas, USA.

[2]Department of Immunology, UT Southwestern Medical Center, Dallas, Texas, USA.

[3]Department of Pharmacy Practice, Texas Tech University Health Science Center, Dallas, Texas, USA.

[4]Department of Internal Medicine, National Taiwan University Hospital, Taipei, Taiwan.

[5]Department of Internal Medicine, Kaohsiung Medical University Hospital, Kaohsiung, Taiwan.

[6]Graduate Institute of Medicine, Kaohsiung Medical University, Kaohsiung, Taiwan.

[7]Quantitative Preclinical & Clinical Sciences Department, Praedicare Inc., Dallas, Texas.

[8]Department of Medicine, University of Cape Town, Observatory, South Africa.

[9]Division of Infectious Diseases and International Health, University of Virginia, USA.

[*]**Corresponding author:**
Shashikant Srivastava, Ph.D.
Department of Pulmonary Immunology, University of Texas Health Centre, 11937 US Highway 271, Tyler, Texas, 75708, USA
Phone: (903) 877-7684
e-mail: Shashi.kant@uthct.edu

Supplemental Figure 1. Changes in the number of viable THP-1 cells in the HFS-*Mkn* upon rifampin treatment.

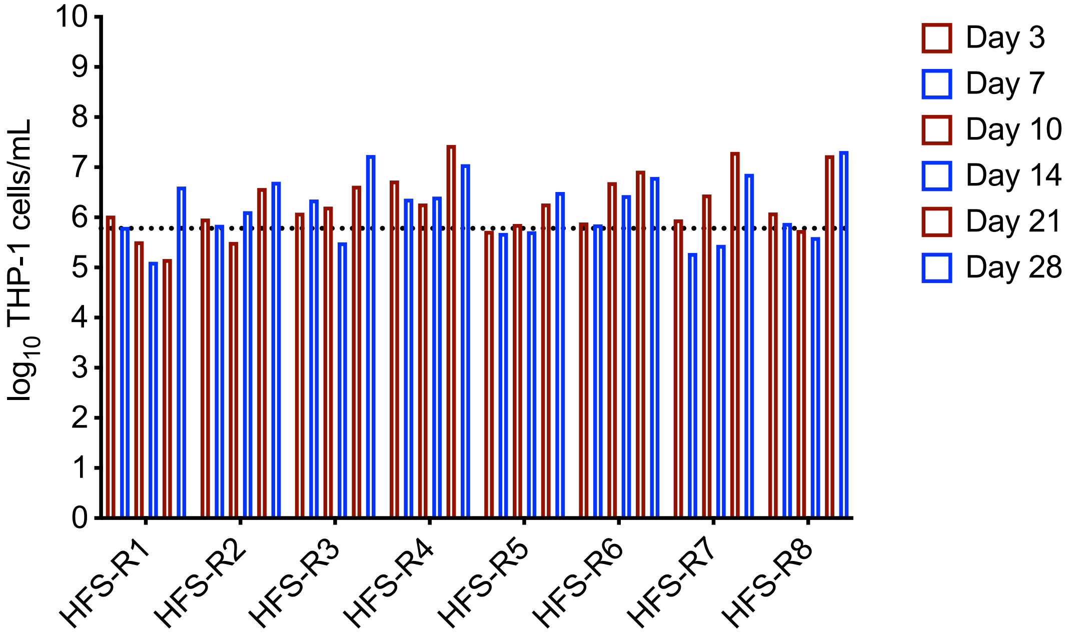

Consistent with the *Mkn* kill seen with different rifampin doses, the number of viable THP-1 cells did not change significantly from baseline to day 28. This indicates intracellular *Mkn* kill by rifampin, hence THP-1 cell survival.

Supplemental Figure 2. Rifampin minimum inhibitory concentration on study day 28.

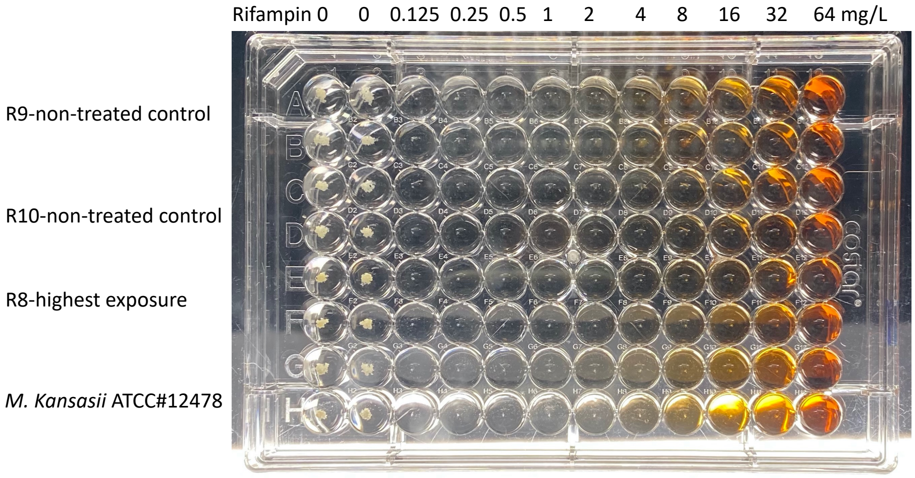

Representative figure for day 28 of the HFS-*Mkn* study. There was no change in the rifampin MIC of the laboratory strain in any HFS-*Mkn* unit, except in the systems with rifampin exposure of fC_{max}/MIC=26.88 where the MIC changed from baseline 0.125 mg/L to 8 mg/L.

Supplemental Figure 3. Time-to-positive as second pharmacodynamics method in the HFS-Mkn study.

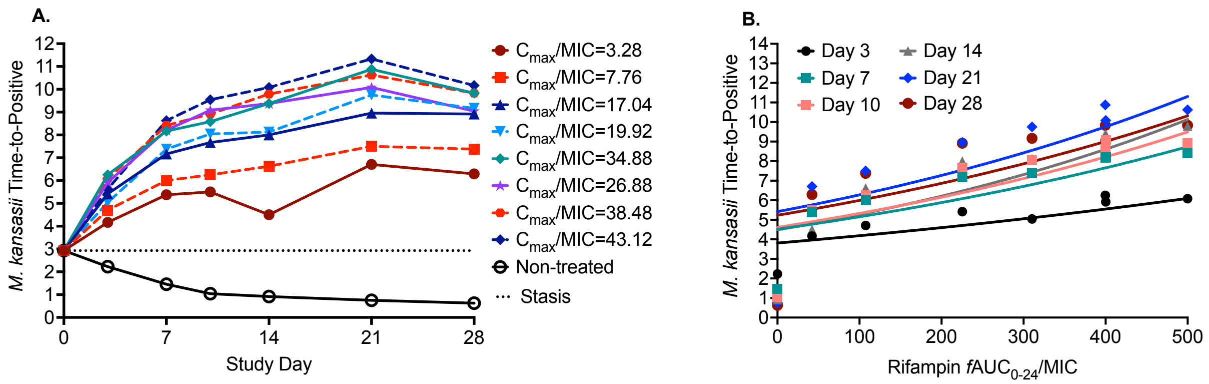

(A) The time-to-positive (TTP) in the non-treated control systems decreased from 2.93 days to 0.625 days by the end of the HFS-*Mkn* study indicating intracellular bacterial growth. Whereas, the TTP in all rifampin treated systems increased indicating bacterial kill. **(B)** Exponential growth model showing relationship between TTP and *Mkn* bacterial burden in the HFS-*Mkn* over time.
